# Concentrations of persistent organic pollutants in maternal plasma and epigenome-wide placental DNA methylation

**DOI:** 10.1186/s13148-020-00894-6

**Published:** 2020-07-13

**Authors:** Marion Ouidir, Pauline Mendola, Germaine M. Buck Louis, Kurunthachalam Kannan, Cuilin Zhang, Fasil Tekola-Ayele

**Affiliations:** 1 Epidemiology Branch, Division of Intramural Population Health Research, Eunice Kennedy Shriver National Institute of Child Health and Human Development, National Institutes of Health, 6710B Rockledge Drive, Bethesda, MD 20892-7004 USA; 2 Office of the Dean, College of Health and Human Services, George Mason University, Fairfax, VA USA; 3 Wadsworth Center, New York State Department of Health, Albany, New York, NY USA; 4 Department of Pediatrics, New York University School of Medicine, New York, NY USA

**Keywords:** Placental DNA methylation, Placental gene expression, Persistent organic pollutants, Polybrominated diphenyl ethers, Polychlorinated biphenyls, Organochlorine pesticides, Poly- and perfluorinated alkyl substances, Epigenome-wide association study, Neonatal anthropometry

## Abstract

**Background:**

Prenatal maternal plasma persistent organic pollutant (POP) concentrations have been associated with neonatal outcomes. However, the underlying mechanisms remain unknown. Placental epigenetic mechanisms may be involved, but no prior epigenome-wide studies have investigated the impact of maternal POPs on placental DNA methylation. We studied the association between maternal plasma POP concentration in early pregnancy and epigenome-wide placental DNA methylation among 260 pregnant women from the NICHD Fetal Growth Studies.

**Results:**

Our analysis focused on POPs with more than 80% plasma concentrations above the limit of quantification, including 3 organochlorine pesticides (hexachlorobenzene, trans-nonachlor, *p,p’*-dichlorodiphenyldichloroethylene), 1 polybrominated diphenyl ether (PBDE 47), 3 polychlorinated biphenyls (138/158, 153, 180), and 6 poly- and perfluorinated alkyl substances (PFASs) (perfluorodecanoic acid, perfluorohexanesulfonic acid, perfluorononanoic acid, perfluorooctanesulfonic acid, perfluoroundecanoic acid (PFUnDA)). Using 5% false discovery rate, POPs were associated with a total of 214 differentially methylated CpG sites (nominal *p* values ranging from 2.61 × 10^−21^ to 2.11 × 10^−7^). Out of the 214 CpG sites, 24 (11%) were significantly correlated with placental expression of 21 genes. Notably, higher PFUnDA was associated with increased methylation at 3 CpG sites (cg13996963, cg12089439, cg18145877) annotated to *TUSC3*, and increased methylation at those 3 CpG sites was correlated with decreased expression of *TUSC3* in the placenta. Increased methylation at cg18145877 (*TUSC3*) and decreased expression of *TUSC3* were correlated with shorter birth length. Out of the 214 CpG sites, methylation at 44 CpG sites was correlated (*p* value < 0.10) with at least one neonatal anthropometry measure (i.e., birth weight, birth length, and head circumference). Seven CpG sites mediated (*p* value < 0.05) the association between PBDE 47 and neonatal anthropometry measures. Genes annotating the top differentially methylated CpG sites were enriched in pathways related to differentiation of embryonic cells (PBDE 47) and in pathways related to brain size and brain morphology (PFASs).

**Conclusions:**

DNA methylation changes in the placenta were significantly associated with maternal plasma POPs concentration. The findings suggest that placental DNA methylation and gene expression mechanism may be involved in the prenatal toxicity of POPs and their association with neonatal anthropometry measures.

## Background

Persistent organic pollutants (POPs) have been used for decades in a large variety of products. Despite the international consensus to reduce or ban these chemicals, exposure persists, mainly through diet, with concentrations still detected in human serum, including in US pregnant women [1] and newborns [2–4]. POPs are ubiquitous endocrine-disrupting compounds (EDC) that interfere with maternal hormones and can impact fetal development and health in later life. Studies have reported that maternal levels of POPs during pregnancy are associated with decrements in fetal growth and birth weight [5–9], neurodevelopmental disorders [10], earlier age of menarche [11], and genitourinary conditions in offspring [12].

The mechanisms underlying the relationships between maternal exposure to these chemicals and fetal outcomes are not yet fully understood. Alterations in placental development have been reported in response to maternal EDC exposure [13], such as modification of the size of the placenta in mice [14], and degeneration of placental trophoblast in rats [15]. Therefore, changes in DNA methylation in the placenta may be one of the potential mechanisms that explain the impact of POPs on human fetal outcomes [16]. Existing studies of POPs and placental DNA methylation were based on candidate gene-based approaches [17–20]. Two studies among 109 pregnant women from the CHECK (Children’s Health and Environmental Chemicals in Korea) cohort reported associations of β-hexachlorocyclohexane (β-HCH) with decreased methylation in *LINE-1* (a surrogate marker of global methylation) and *p*,*p*′-dichlorodiphenyltrichloroethane (*p*,*p*′-DDT) with increased methylation of *IGF2* (implicated in placental and fetal growth) [17] and MCT8 among boys [18]. *P,p*′-dichlorodiphenyldichloroethylene (*p,p*′-DDE) and polybrominated diphenyl ether-47 (PBDE 47) were significantly associated with increased methylation in *DIO3* among female infants [18]. Higher PBDE 66 in cord blood was associated with decreased placental methylation in *LINE-1* and higher PBDEs 153 and 209 with decreased placental methylation of *IGF2* among Chinese [20] while others did not find any associations [19]. Most studies have investigated individual POPs, although pregnant women are exposed to a mixture of chemicals [1]. In this study, we assessed placental DNA methylation related to individual POPs and to chemical classes (i.e., sum of POPs in each chemical class).

We performed an epigenome-wide association study (EWAS) to identify placental DNA methylation associated with maternal plasma concentration of POPs in early gestation (10 weeks 0 days to 13 weeks 6 days) among 260 pregnant women participating in the *Eunice Kennedy Shriver* National Institute of Child Health and Human Development’s (NICHD) Fetal Growth Studies–Singletons cohort (which comprised 2802 pregnant women from 12 clinic sites within the USA). Genes annotated to the differentially methylated CpG sites were tested for enrichment of molecular pathways. We assessed correlations between DNA methylation at the POP-associated CpG sites and placental expression of the annotated genes. Further, to evaluate the relevance of the differentially methylated CpG sites to fetal growth, we examined correlations of methylation and gene expression with birth weight, birth length, and head circumference. Lastly, we investigated the potential mediating pathway from POPs to neonatal anthropometry measures via placental DNA methylation at the POP-associated CpG sites that were correlated with neonatal anthropometry measures (i.e., birth weight, birth length, and head circumference).

## Results

### Study population

Characteristics of the 260 women included in the analysis are presented in Table 1. The mean (sd) age and pre-pregnancy BMI were 27.6 (5.2) years and 23.3 (2.9) kg/m^2^, respectively, and 126 (48.5%) were nulliparous (Table 1). There was no difference in characteristics of women included in our analytic sample and the full NICHD Fetal Growth Study cohort (Supplementary Table S1). Our analysis included chemicals with more than 80% plasma POP concentrations above the limit of quantification (LOQ). These included 7 persistent lipophilic chemicals: 3 organochlorine pesticides (OCPs: hexachlorobenzene (HCB), trans-nonachlor, *p,p′*-DDE), 1 polybrominated diphenyl ethers (PBDE 47) and 3 polychlorinated biphenyls (PCB congeners 138/158, 153, 180), and 6 persistent non-lipophilic chemicals: poly- and perfluorinated alkyl substances (PFASs: perfluorodecanoic acid (PFDA), perfluorohexanesulfonic acid (PFHxS), perfluorononanoic acid (PFNA), perfluorooctanoic acid (PFOA), perfluorooctanesulfonic acid (PFOS), perfluoroundecanoic acid (PFUnDA)) (Supplementary Table S2).

**Table 1:** Characteristics of the study sample from the NICHD fetal Growth Studies–Singletons (*n* = 260)

		Mean ± SD or ***N*** (%) or Median [p25-p75]
**Maternal age, years**		27.6 ± 5.2
**Gestational age at enrollment, weeks**		12.7 ± 0.9
**Maternal pre-pregnancy BMI, kg/m**^**2**^		23.3 ± 2.9
**Maternal race/ethnicity**		
Non-Hispanic White		67 (25.8)
Non-Hispanic Black		59 (22.7)
Hispanic		86 (33.1)
Asians		48 (18.5)
**Parity**		
Nulliparous		126 (48.5)
Parous		134 (51.5)
**Infant sex**		
Male		130 (50.0)
Female		130 (50.0)
**Maternal POP plasma concentration**		
**Chemical class**	**Chemicals**	
OCPs (ng/g)	HCB	6.28 [3.50, 10.10]
	Trans-nonachlor	4.25 [2.48, 7.35]
	*p,p′*-DDE	85.26 [54.09, 218.81]
	Σ OCPs	98.83 [65.70, 228.20]
PBDEs (ng/g)	PBDE 47	8.64 [3.60, 16.67]
PCBs (ng/g)	PCB 138/158	5.03 [2.78, 8.17]
	PCB 153	5.74 [3.18, 10.09]
	PCB 180	3.30 [1.90, 5.25]
	Σ PCBs	14.13 [8.06, 23.24]
PFASs (ng/mL)	PFDA	0.24 [0.16, 0.42]
	PFHxS	0.70 [0.43, 1.27]
	PFNA	0.75 [0.54, 1.22]
	PFOA	2.20 [1.30, 3.07]
	PFOS	4.74 [3.20, 7.65]
	PFUnDA	0.16 [0.09, 0.32]
	Σ PFASs	9.33 [6.39, 14.09]

All POP concentrations were based upon machine-measured concentrations without substitution of concentrations < LOQ. OCPs, PCBs, and PBDEs concentrations are adjusted for total plasma lipids. Missing data have been imputed

*POP* persistent organic pollutant

### Epigenome-wide analyses

**Fig. 1:**
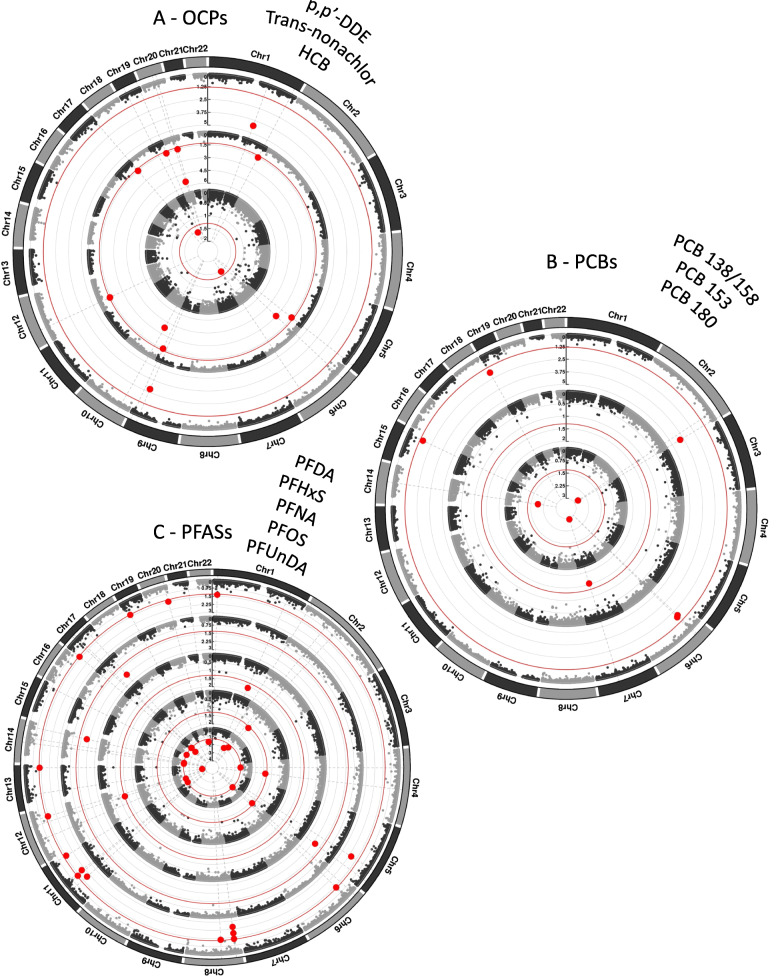
Manhattan plot of associations between maternal plasma concentrations of POPs and DNA methylation in placenta. Adjusted for maternal self-reported race/ethnicity, maternal age in years, fetal sex, maternal pre-pregnancy BMI, cotinine level, total lipids (except PFASs),methylation sample plate, first three methylation principal component (PCs), the first 10 genotype PCs, and surrogate variable analysis (SVA)

**Fig. 2:**
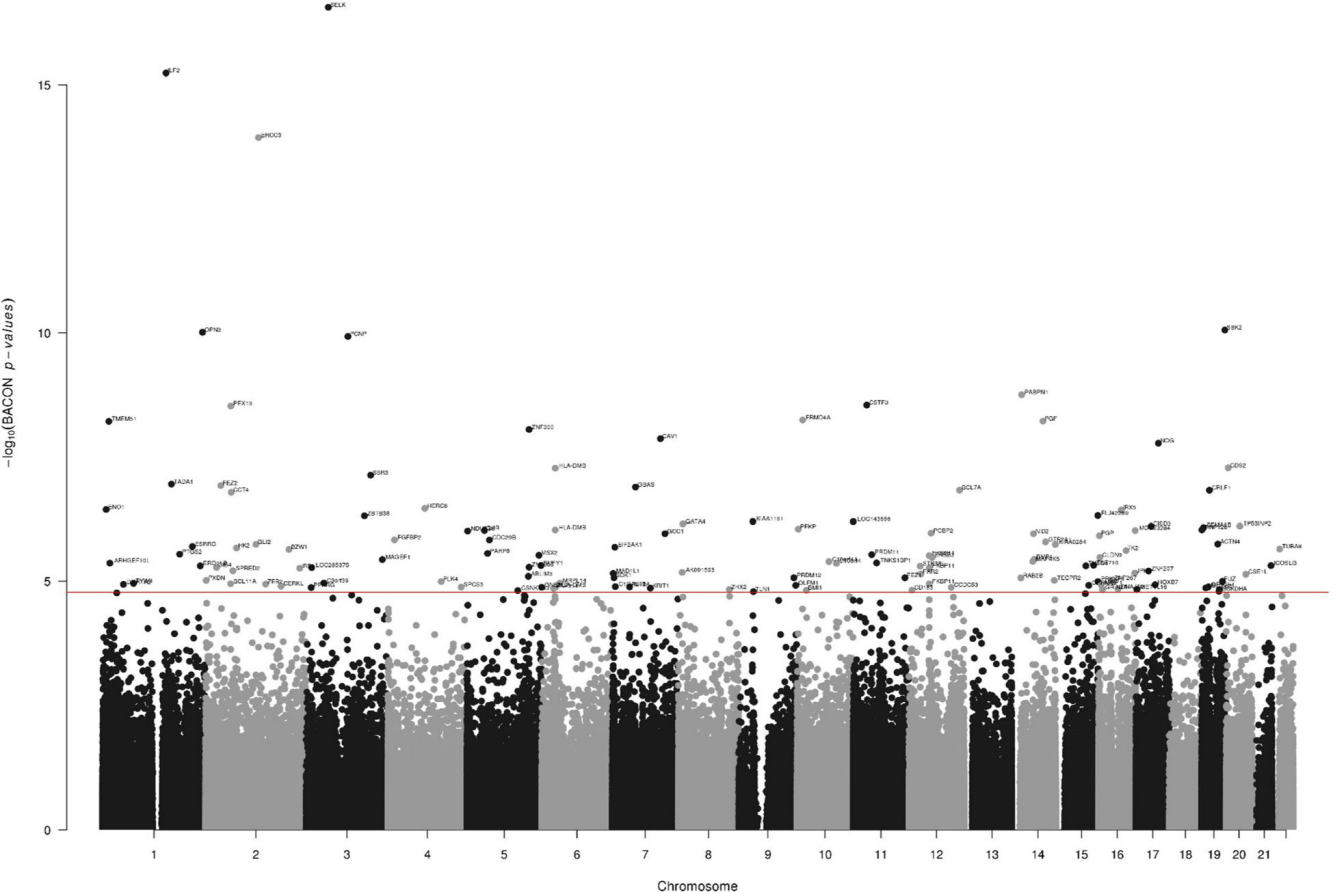
Manhattan plot of associations between maternal plasma concentrations of PBDE 47 and DNA methylation in placenta. Adjusted for maternal self-reported race/ethnicity, maternal age in years, fetal sex, maternal pre-pregnancy BMI, cotinine level, total lipids, methylation sample plate, first three methylation principal component (PCs), the first 10 genotype PCs, and surrogate variable analysis (SVA)

In total, maternal early pregnancy plasma concentrations of POPs were significantly associated with placental DNA methylation at 214 CpG sites annotated to 205 genes (BACON-corrected false discovery rate (FDR) *p* values < 0.05, nominal *p* values ranging from 2.61 × 10^−21^ to 2.11 10^−7^, Supplementary Table S3). The majority of the differentially methylated CpG sites (49.9%) were located in CpG island regions (Supplementary Figure S1). OCPs (i.e., HCB, trans-nonachlor, and *p,p′*-DDE) were associated with methylation at 14 CpG sites. The smallest association *p* value and highest strength of association was between trans-nonachlor and cg27641830 (*RBM39*, *β* = − 7.98, 95% confidence interval (CI) − 10.47 to − 5.48, FDR *p* value = 3.71 × 10^−6^; Table 2, Fig. 1). PBDE 47 was associated with methylation at 133 CpG sites, the smallest association *p* value being with cg06801544 (*SELK*; FDR *p* value = 1.12 × 10^−11^; Fig. 2). The highest strength of association was with cg19595912 (*ERO1LB*, *β* = − 0.84, 95% CI − 1.13 to − 0.55). All measured PCBs (congeners 138/158, 153, and 180) were associated with methylation at 9 CpG sites with the smallest association *p* value being between PCB 180 and cg18663897 (*NDUFA10*; FDR *p* value = 8.27 × 10^−5^). The highest strength of association was between PCB 138/158 and cg02537221 (*NHEJ1*, *β* = − 0.68, 95% CI − 0.88 to − 0.49). PFASs (i.e., PFDA, PFHxS, PFNA, PFOS, and PFUnDA) were associated with 39 CpG sites, of which the smallest association *p* value being between PFDA and cg04117229 (*SPG20*; FDR *p* value = 2.69 × 10^−4^; Table 3). The highest strength of association was between PFUnDA and cg24298878 (*ILF3*, *β* = − 1.22, 95% CI − 1.67 to − 0.78). Notably, higher PFUnDA concentration was associated with increased methylation at 3 differentially methylated CpG sites annotated to *TUSC3* (cg13996963, cg12089439, and cg18145877). Analyses of chemical classes (i.e., sum of OCPs, sum of PCBs, and sum of PFASs) found that the sum of OCPs was associated with 25 CpG sites, the smallest association *p* value being with cg26773954 (*TEX29*; FDR *p* value = 4.34 × 10^−6^; Supplementary Table S3). The highest strength of association was with cg26605427 (*MGC23284*, *β* = − 1.80, 95% CI − 2.26 to − 1.33). The sum of PCBs was associated with 2 CpG sites, the smallest association *p* value being with cg06219267 (*FBXO24*; FDR *p* value = 0.018), while the highest strength of association was with cg02537221 (*NHEJ1*, *β* = − 0.64, 95% CI − 0.84 to − 0.45).

**Table 2:** OCPs and PCBs—significant adjusted difference (BACON-corrected FDR *p* values < 0.05) in placenta DNA methylation associated with maternal plasma concentration of POPs (*n* = 260)

Class	Chemicals	CpG	Gene	logFC	[95% CI]	BACON-corrected ***p*** value	BACON-corrected FDR ***p*** value
**OCPs**	HCB	cg27066638	*GMIP*	− 7.98	[− 10.47, − 5.48]	6.70E−08	2.74E−02
		cg11792277	*C6orf217*	1.27	[0.87, 1.67]	1.69E−07	3.46E−02
	Trans-nonachlor	cg27641830	*RBM39*	− 5.52	[− 6. 18, − 4.24]	9.07E−12	3.71E−06
		cg04689409	*THNSL1*	− 2.76	[− 3.47, − 2.05]	8.13E−10	1.66E−04
		cg19851715	*HIST1H2BI*	0.60	[0.44, 0.77]	7.14E−09	9.73E−04
		cg00718518	*SH3PXD2B*	− 0.26	[− 0.34, − 0.18]	3.23E−07	3.04E−02
		cg19358877	*ZNF471*	− 2.47	[− 3.52, − 1.69]	4.52E−07	3.04E−02
		cg13406593	QRFP*	− 0.29	[− 0.39, − 0.20]	5.14E−07	3.04E−02
		cg17462356	*FASN*	− 2.76	[− 3.64, − 1.89]	5.75E−07	3.04E−02
		cg23330710	*PIGT*	− 1.37	[− 1.81, − 0.94]	5.95E−07	3.04E−02
		cg07814876	*GGPS1;ARID4B*	− 2.18	[− 2.88, − 1.49]	6.73E−07	3.06E−02
		cg04568710	*ALG10B*	− 0.95	[− 1.26, − 0.64]	1.07E−06	4.37E−02
	p,p′-DDE	cg24596729	LMX1A*	0.13	[0.10, 0.16]	1.27E−10	5.20E−05
		cg13910813	*AKNA*	− 0.26	[− 0.33, − 0.19]	7.74E−09	1.58E−03
**PCBs**	PCB 138/158	cg06219267	*FBXO24*	− 0.63	[− 0.81, − 0.46]	2.95E−09	1.21E−03
		cg02537221	*NHEJ1*	− 0.68	[− 0.88, − 0.49]	8.82E−09	1.80E−03
		cg01110147	*PSMA3*	0.23	[0.16, 0.29]	9.17E−08	1.25E−02
	PCB 153	cg06219267	*FBXO24*	− 0.63	[− 0.81, − 0.44]	7.04E−08	2.88E−02
	PCB 180	cg18663897	*NDUFA10*	− 0.56	[− 0.70, − 0.42]	2.02E−10	8.27E−05
		cg19179910	*MUC21*	− 0.45	[− 0.57, − 0.32]	5.25E−08	1.07E−02
		cg11562147	*C6orf222*	0.11	[0.08, 0.14]	1.36E−07	1.39E−02
		cg26184687	*MKNK2*	− 0.18	[− 0.23, − 0.12]	1.11E−07	1.39E−02
		cg17874528	*KLHL25*	− 0.23	[− 0.30, − 0.16]	3.21E−07	2.63E−02

Adjusted for maternal self-reported race/ethnicity, maternal age in years, fetal sex, maternal pre-pregnancy BMI, total lipid, cotinine level, methylation sample plate, first three methylation principal component (PCs), the first 10 genotype PCs, and surrogate variable analysis (SVA)

*logFC* logarithm of fold change, *FDR* false discovery rate

* Annotation from the nearest gene

**Table 3:** PFASs—significant adjusted differences (BACON-corrected FDR *p* values < 0.05) in placenta DNA methylation associated with maternal plasma concentration of PFASs (*n* = 262)

Chemicals	CpG	Gene	logFC	[95% CI]	BACON-corrected ***p*** value	BACON-corrected FDR ***p*** value
PFDA	cg04117229	*SPG20*	− 0.61	[− 0.78, − 0.45]	6.57E−10	2.69E−04
	cg23851558	*LOC400940*	− 0.16	[− 0.20, − 0.11]	4.83E−08	9.88E−03
	cg14355889	*MYO18A*	− 0.07	[− 0.09, − 0.05]	7.36E−08	1.00E−02
	cg09441069	*FTCD*	− 0.09	[− 0.12, − 0.06]	2.46E−07	2.30E−02
	cg06858599	*SNRPG*	− 0.16	[− 0.22, − 0.11]	2.81E−07	2.30E−02
	cg13746315	MCTP2*	− 1.05	[− 1.41, − 0.69]	8.39E−07	4.80E−02
	cg01352468	*ANKRD13B*	− 0.31	[− 0.41, − 0.20]	9.19E−07	4.80E−02
	cg07471462	*SLC39A7*	− 0.21	[− 0.28, − 0.14]	1.11E−06	4.80E−02
	cg23317906	*TMX1*	− 0.15	[− 0.20, − 0.10]	1.20E−06	4.80E−02
	cg18844176	*MTHFD1*	− 0.33	[− 0.45, − 0.22]	1.21E−06	4.80E−02
	cg08912652	*SNX19*	− 0.33	[− 0.44, − 0.21]	1.41E−06	4.80E−02
	cg10391212	*CORIN*	0.06	[0.04, 0.08]	1.41E−06	4.80E−02
	cg13006259	*MYL6B*	− 0.63	[− 0.85, − 0.41]	1.64E−06	4.80E−02
	cg07169834	*RDM1*	0.11	[0.07, 0.15]	1.55E−06	4.80E−02
PFHxS	cg21058927	*CISD2*	− 0.54	[− 0.71, − 0.37]	1.06E−07	4.12E−02
	cg11428546	*AFF3*	− 0.04	[− 0.05, − 0.03]	2.39E−07	4.12E−02
	cg10530492	*NOL7*	− 0.12	[− 0.16, − 0.08]	3.02E−07	4.12E−02
PFNA	cg26808417	*ARL8A*	− 0.10	[− 0.13, − 0.07]	6.00E−08	2.45E−02
	cg21502999	*ELK3*	0.31	[0.21, 0.41]	2.40E−07	4.91E−02
PFOS	cg17921248	*PRKCA*	0.06	[0.05, 0.08]	1.43E−08	5.84E−03
	cg11891579	*EBF1*	0.16	[0.11, 0.21]	4.53E−08	9.27E−03
	cg01954404	SERPINA1*	− 0.10	[− 0.14, − 0.07]	7.22E−08	9.85E−03
PFUnDA	cg13996963	*TUSC3*	0.39	[0.27, 0.51]	8.83E−09	3.61E−03
	cg12599971	GRAMD3*	− 0.42	[− 0.55, − 0.29]	4.49E−08	9.18E−03
	cg15719126	*MAPK8IP1*	− 0.22	[− 0.29, − 0.15]	8.72E−08	1.10E−02
	cg13705616	*SHANK2*	0.18	[0.12, 0.24]	1.07E−07	1.10E−02
	cg12089439	*TUSC3*	0.40	[0.27, 0.53]	1.92E−07	1.37E−02
	cg18145877	*TUSC3*	0.63	[0.42, 0.84]	2.01E−07	1.37E−02
	cg08912652	*SNX19*	− 0.41	[− 0.55, − 0.27]	4.91E−07	2.87E−02
	cg03223126	*CUX2*	− 0.34	[− 0.46, − 0.22]	7.32E−07	3.34E−02
	cg16347018	*ATP5E*	− 1.10	[− 1.49, − 0.72]	7.35E−07	3.34E−02
	cg14067524	*MYO16*	− 0.29	[− 0.39, − 0.18]	1.13E−06	4.38E−02
	cg10058241	*DOC2B*	0.17	[0.11, 0.23]	1.32E−06	4.38E−02
	cg09442654	*FAM150A*	0.55	[0.35, 0.75]	1.40E−06	4.38E−02
	cg07471462	*SLC39A7*	− 0.24	[− 0.33, − 0.15]	1.50E−06	4.38E−02
	cg10550693	*SYVN1*	0.07	[0.04, 0.09]	1.43E−06	4.38E−02
	cg24722365	*KIF1B*	− 0.14	[− 0.19, − 0.09]	1.61E−06	4.38E−02
	cg18336854	*TUSC3*	− 0.18	[− 0.24, − 0.11]	1.79E−06	4.58E−02
	cg24298878	*ILF3*	− 1.22	[− 1.67, − 0.78]	1.98E−06	4.76E−02

Adjusted for maternal self-reported race/ethnicity, maternal age in years, fetal sex, maternal pre-pregnancy BMI, cotinine level, methylation sample plate, first three methylation principal component (PCs), the first 10 genotype PCs and Surrogate Variable Analysis (SVA).

*logFC* logarithm of fold change, *FDR* false discovery rate

*Annotation from the nearest gene

Analysis of differentially methylated regions (DMR) found that PBDE 47 was associated with three DMRs, the smallest association *p* value being the DMR annotated to *HLA-DMB* (FDR *p* value = 3.43 × 10^−17^), and highest strength of association being annotated to *ZNF300* (*β* = −0.28, 95% CI − 0.33 to − 0.22); PFUnDA was associated with one DMR annotated to *TUSC3* (*β* = 0.40, 95% CI 0.30 to 0.49, FDR *p* value = 2.51 × 10^−10^, Supplementary Table S4).

### Correlations between DNA methylation and gene expression

Out of the 214 differentially methylated CpG sites, 24 CpG sites were correlated with placental gene expression of 21 unique genes (*p* values < 0.05, Table 4, Supplementary Table S5). The strongest correlation was between cg12089439 and expression of *TUSC3* (*r* = − 0.55, *p* value = 3.70 × 10^−6^). Further examination of the correlations between individual POP concentrations and gene expression levels found that higher maternal trans-nonachlor concentration was marginally correlated (*r* = − 0.25, *p* value = 0.05) with decreased expression of *SH3PXD2B* (Supplementary Table S6)*.* This is consistent with our DNA methylation analysis finding where higher maternal plasma concentration of trans-nonachlor was associated with decreased methylation at cg00718518 (*SH3PXD2B*) (Table 2), and lower methylation at cg00718518 was correlated with decreased expression of *SH3PXD2B* (Table 4). *SH3PXD2B* displayed the highest expression in female reproductive tissues (Supplementary Figure S4).

**Table 4:** Significant correlations between DNA methylation at the top differentially methylated CpG sites and gene expression in placenta (*n* = 62)

Chemicals	CpG site	Gene	Spearman correlation	***p*** value
Trans-nonachlor	cg00718518	*SH3PXD2B*	0.343	0.006
	cg04689409	*THNSL1*	0.265	0.037
Σ OCPs	cg21733927	*SEPT9*	− 0.286	0.024
	cg26314399	*PLEKHG4B*	0.252	0.048
PBDE 47	cg23083936	*BCKDHA*	0.311	0.014
	cg04848823	*C16orf72*	0.258	0.043
	cg23880581	*CHRNA3*	0.382	0.002
	cg05883907	*FKBP11*	− 0.369	0.003
	cg08288330	*FUZ*	− 0.369	0.003
	cg12562660	*GLI2*	− 0.251	0.049
	cg11079619	*INHBA*	− 0.448	0.000
	cg14190975	*OLFM1*	0.267	0.036
	cg24801210	*PCNP*	0.288	0.023
	cg27272487	*SPRED2*	0.254	0.046
	cg26538590	*TK2*	0.290	0.022
	cg06869505	*TMEM51*	− 0.304	0.016
	cg04675542	*ZNF300*	− 0.348	0.006
	cg02343823	*ZNF300*	− 0.369	0.003
PFDA	cg09441069	*FTCD*	0.549	3.78E−06
PFOS	cg11891579	*EBF1*	− 0.308	0.015
PFUnDA	cg16347018	*ATP5E*	0.259	0.042
	cg12089439	*TUSC3*	− 0.550	3.70E−06
	cg18145877	*TUSC3*	− 0.418	0.001
	cg13996963	*TUSC3*	− 0.416	0.001

### Canonical pathways

The genes mapping the differentially methylated CpG sites were enriched in Ingenuity Pathway Analysis (IPA) canonical disease and function pathways (Supplementary Table S7). Genes annotating the CpG sites differentially methylated with increasing levels of PBDE 47 were enriched in disease and functional annotation pathways including differentiation of embryonic cells (*p* value = 5.52 × 10^−7^). Genes annotating CpG sites associated with PFAS exposure were enriched in disease and functional annotation pathways, including size of the brain (*p* value = 3.39 × 10^−4^) and morphologies of the central nervous system (*p* value = 2.41 × 10^−4^), brain (*p* value = 9.75 × 10^−4^), and head (*p* value = 1.70 × 10^−3^). The top IPA canonical pathways included Estrogen Receptor Signaling (*ATP5F1E*, *MYL6B*, *PRKCA*) and ERK/MAPK signaling (*ELK3*, *PRKCA*), both enriched for PFASs (Supplementary Table S8), and the top IPA canonical networks were enriched in cellular and tissue development (Supplementary Table S9).

### Functional annotation analyses

To understand whether single nucleotide polymorphisms (SNPs) influence DNA methylation of the top-associated CpG sites, we assessed methylation quantitative trait loci (*cis*-meQTLs) within 1 mega base distance from the differentially methylated CpG sites in published databases of meQTLs in the placenta [21, 22] and blood (including cord blood and peripheral blood samples from whole blood, buffy coats, white blood cells, and blood spots) [23]. In the placenta, cg12599971 (*GRAMD3*) and cg18145877 (*TUSC3*) that were associated with PFUnDA concentration in our study have been reported to be *cis*-meQTL targets for SNPs in *GRAMD3* and *TUSC3* (Supplementary Table S10). Both genes displayed the highest expression in the placenta (Supplementary Figure S3). In the blood, 22 CpG sites that were associated with trans-nonachlor, PBDE 47, PFDA, and PUnDA in our study have been reported to be *cis*-meQTL targets for 3831 unique SNPs located in/near 104 genes (Supplementary Table S11), including *SH3PXD2B* and *TUSC3*.

### Correlations between top CpG sites and neonatal anthropometry measures

**Fig. 3:**
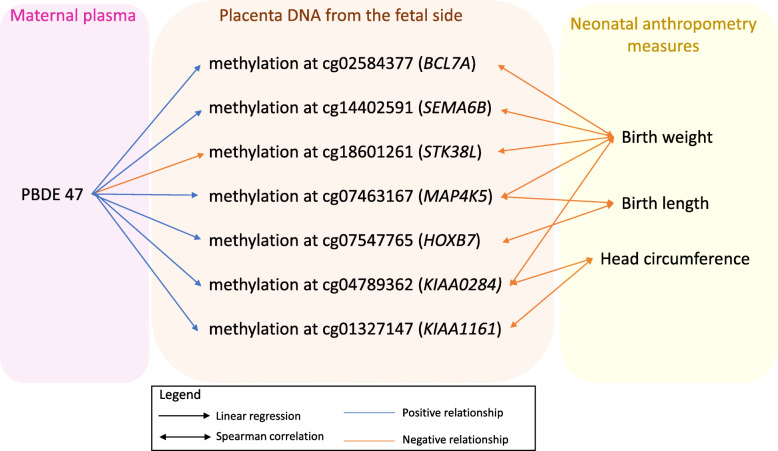
Correlation and covariate-adjusted mediation analysis between PBDE 47, differentially methylated CpG sites, and neonatal anthropometry

Previously, we found significant associations between maternal plasma concentrations of specific POPs and fetal growth and birth anthropometry measures [8, 9]. To examine whether the change in methylation at the CpG sites significantly associated with POPs in the present analysis are related to neonatal anthropometry, we tested the correlations of methylation levels at each of the 214 CpG sites with neonatal anthropometry measures (birth weight, birth length, and head circumference). Among them, 44 CpG sites were correlated with at least one neonatal anthropometry measure (Supplementary Table S12). We conducted mediation analysis to investigate whether placental DNA methylation at those 44 CpG sites was in the pathway between POPs and neonatal anthropometry. Five CpG sites (cg02584377 (*BCL7A*), cg14402591 (*SEMA6B*), cg18601261 (*STK38L*), cg07463167 (*MAP4K5*), and cg04789362 (*KIAA0284*)) significantly mediated the inverse association between PBDE 47 and birthweight (Table 5). In addition, two CpG sites (cg01327147 (*KIAA1161*) and cg04789362 (*KIAA0284*)) significantly mediated the inverse association between PBDE 47 and head circumference. PBDE 47 was negatively correlated with birth weight (*r* = − 0.16, *p* value = 0.01) and head circumference (*r* = − 0.16, *p* value = 0.01) (Fig. 3).

**Table 5:** Spearman correlation and covariate-adjusted mediation analysis between top differentially methylated CpG sites and neonatal anthropometry

Chemicals	ProbeID	Gene name	Birth weight				Birth length				Head circumference			
			Corr.	Mediation analysis			Corr.	Mediation analysis			Corr.	Mediation analysis		
			*r*	ACME	ADE	TE	*r*	ACME	ADE	TE	*r*	ACME	ADE	TE
Σ OCPs	cg21733927	*SEPT9*	0.15*	- 32.96	123.97	91.01	0.21**	− 0.08	0.06	− 0.02	0.17**	− 0.08	0.33	0.26
	cg11938455	*DTNBP1*	0.23***	− 101.84****	192.51***	90.67	0.19**	− 0.45****	0.43	− 0.02	0.18**	− 0.18*	0.43*	0.25
	cg06510261	*GCET2*	0.1				0.12				0.15*	− 0.15**	0.41*	0.26
	cg08704611	*HEXDC*	− 0.16*	24.73*	65.47	90.2	0				− 0.09			
	cg00045303	*SETD4*	− 0.11				0.07				− 0.14 *	0.05	0.21	0.26
	cg02303677	*TACR2*	0.17**	54.32**	37.71	92.02	0.14*	0.16	- 0.19	- 0.03	0.18**	0.06	0.2	0.25
PBDE 47	cg02584377	*BCL7A*	− 0.15*	− 17.72***	17.13	− 0.6	− 0.06				− 0.07			
	cg21237837	*BMP4*	− 0.14*	13.76	− 15.6	− 1.84	− 0.01				− 0.01			
	cg18209470	*CAV1*	− 0.09				− 0.18**	− 0.03	0.08	0.05	− 0.09			
	cg03621974	*CD163*	− 0.16*	7.62	− 8.61	− 0.99	− 0.22***	0.06	− 0.01	0.05	− 0.14*	0.02	0.03	0.05
	cg10492999	*CLDN9*	− 0.15*	− 8.07	6.77	− 1.3	− 0.12				− 0.17**	− 0.03	0.09	0.06
	cg11464615	*CRLF1*	0.07				0.07				0.15*	− 0.03	0.08	0.06
	cg25851789	*ESRRG*	0.09				0.14*	− 0.07	0.12	0.05	0.03			
	cg13979581	*GCC1*	− 0.16*	3.94	− 5.2	− 1.26	− 0.07				− 0.06			
	cg22306009	*GNB2L1*	0.14*	2	− 3.02	− 1.02	0.1				0.25***	0.01	0.05	0.05
	cg05089296	*GTF2A1*	− 0.14*	− 9.24	7.75	− 1.49	− 0.15*	− 0.06*	0.12	0.06	− 0.1			
	cg08308032	*HK2*	0.11				0.1				0.14*	0	0.05	0.05
	cg10123514	*HLA-DMB*	0.08				0.07				0.15*	0.02	0.04	0.06
	cg13524037	*HLA-DMB*	0.03				− 0.01				0.21**	0.02	0.03	0.05
	cg07547765	*HOXB7*	− 0.15*	− 6.67	5.28	− 1.39	− 0.14 *	− 0.06**	0.11	0.05	− 0.09			
	cg02087289	*ICOSLG*	0.03				0.05				0.14*	0	0.05	0.05
	cg04789362	*KIAA0284*	− 0.17**	− 19.27**	18.38	− 0.89	− 0.16 *	− 0.09*	0.14	0.06	− 0.16 *	− 0.08**	0.13	0.06
	cg01327147	*KIAA1161*	− 0.11				− 0.08				− 0.15*	− 0.07**	0.12	0.05
	cg07463167	*MAP4K5*	− 0.28****	− 21.71***	19.81	− 1.89	− 0.22**	− 0.11***	0.17	0.06	− 0.03			
	cg14827832	*METTL16*	0.11				0.16*	− 0.08*	0.14	0.05	0.22***	− 0.03	0.09	0.06
	cg13592399	*NID2*	− 0.16*	6.64	− 8.13	− 1.49	− 0.13				− 0.12			
	cg08405284	*OR10H1*	0.18 **	0.1	− 2.31	− 2.21	0.12				0.14			
	cg24604417	*PARP8*	0.12				0.2**	− 0.06	0.12	0.06	0.19**	− 0.05	0.11	0.05
	cg24877391	*PEX13*	0.04				0.15*	− 0.03	0.09	0.06	0.07			
	cg24192328	*PGF*	0.11				0.22***	− 0.03	0.08	0.05	0.14*	− 0.02	0.08	0.06
	cg02085815	*PLK4*	0.13				0.17**	− 0.01	0.06	0.05	0.07			
	cg24715445	*RNF126*	0.1				0.08				0.16 *	0.04	0.01	0.05
	cg25161252	*SBK2*	0.15*	− 2.51	0.71	− 1.8	0.16*	− 0.02	0.07	0.05	0.19**	− 0.03	0.08	0.05
	cg14402591	*SEMA6B*	− 0.16*	− 20.81**	19.53	− 1.28	− 0.06				− 0.06			
	cg00559054	*SSR3*	0.08				0.19**	− 0.04	0.1	0.06	− 0.02			
	cg18601261	*STK38L*	− 0.19**	38.9****	− 40.14	− 1.24	− 0.1				− 0.08			
	cg26538590	*TK2*	− 0.19**	3.36	− 5.22	− 1.87	− 0.13				− 0.11			
	cg06869505	*TMEM51*	− 0.02				0.14*	− 0.04	0.09	0.05	− 0.03			
	cg17719053	*TYW3*	− 0.11				− 0.16*	− 0.03	0.09	0.06	− 0.06			
	cg19530176	*ZBTB38*	0.09				0.14*	− 0.04	0.1	0.06	0.08			
PCB 138_158	cg06219267	*FBXO24*	− 0.03				0.18**	− 0.2*	0.26	0.06	− 0.09			
	cg01110147	*PSMA3*	0.24***	− 0.61	24.39	23.78	0.15*	− 0.12*	0.19	0.07	0.18**	0	0.06	0.05
PCB153	cg06219267	*FBXO24*	− 0.03				0.18**	− 0.27*	0.34	0.06	− 0.09			
Σ PCBs	cg06219267	*FBXO24*	− 0.03				0.18**	− 0.25**	0.36	0.11	− 0.09			
PFDA	cg09441069	*FTCD*	0.12				0.08				0.22***	− 0.03	− 0.09	− 0.12
	cg23851558	*LOC400940*	0.09				0.17**	− 0.04	− 0.18	− 0.21	0.12			
	cg18844176	*MTHFD1*	− 0.03				0.15*	− 0.06	− 0.15	− 0.22	− 0.16**	0.04	− 0.16	− 0.12
	cg13006259	*MYL6B*	− 0.15*	15.13	− 56.43	− 41.31	− 0.11				0.05			
PFUnDA	cg24722365	*KIF1B*	0.07				0.17**	0.01	− 0.26	− 0.25	0.05			
	cg15719126	*MAPK8IP1*	− 0.22***	13.35	− 69.97	− 56.62	− 0.18**	0.06	− 0.33	− 0.27	− 0.18**	0.03	− 0.07	− 0.04
	cg14067524	*MYO16*	− 0.15*	0.08	− 58.53	− 58.45	0.03				− 0.04			
	cg18145877	*TUSC3*	− 0.14				− 0.17**	− 0.01	− 0.25	− 0.26	− 0.11			

*Corr*. Spearman correlation, *ACME* average causal mediation effects, *ADE* average direct effect, *TE* total effect

P-values: * < 0.10; **p < 0.05; ***p < 0.01; ****p < 0.001 Adjusted for maternal self-reported race/ethnicity, maternal age in years, fetal sex, maternal pre-pregnancy BMI, cotinine level, total lipids (except PFASs), methylation sample plate, first three methylation principal component (PCs), and the first 10 genotype PCs

Out of the 21 genes for which expression levels were correlated with DNA methylation at the top-significant CpG sites, higher expressions of *TUSC3* were positively correlated with increased neonatal length (*r* = 0.26, *p* value = 0.04) (Supplementary Table S13).

## Discussion

In this study, we assessed the relation between maternal plasma POP concentrations during early pregnancy and genome-wide DNA methylation in the placenta. Specific maternal plasma POP concentrations were associated with 214 differentially methylated CpG sites. Of the 214 CpG sites, 24 were correlated with placental expression of the annotated genes. We found strong evidence for association of maternal plasma PFUnDA concentration with *TUSC3* based on consistent findings from DNA methylation, gene expression, and meQTL analyses. Similarly, we found consistent evidence from DNA methylation and gene expression data on the impact of maternal plasma concentration of trans-nonachlor on *SH3PXD2B*. The correlations between DNA methylation at the POPs-associated CpG sites and neonatal anthropometry suggest that placental epigenetic mechanisms may underlie the influence of specific maternal plasma POP concentrations on fetal growth.

Two differentially methylated CpG sites identified in the present study have been associated with exposure to chemicals in previous EWAS analyses: cg02343823 (*ZNF300)* associated with PBDE 47 in our study has been associated with polybrominated biphenyl in adult blood [24], and cg18145877 (*TUSC3*) associated with PFUnDA in our study has been associated with PCBs in peripheral blood leucocytes [25]. Furthermore, CpG sites annotated to the same genes though different loci have previously been associated with blood levels of chemicals (Supplementary Table S14). For example, *CUX2*, *FAM150A*, and *FTCD*-annotated CpG sites were associated with specific PFASs in our study as was PFAS concentrations in cord blood [26]. Many genes identified as being associated with maternal plasma concentration of PBDE 47 in our study have been associated with polybrominated biphenyl in adult blood (i.e., *HLA-DM*, *CIB4*, *ARHGEF10L*, *BCL11A*, *CDS2*, *ENO1*, *HOXB7*, *INHBA*, *PCBP2*, *SDK1*, *SPRED2*, *ZBTB38*, *ZEB2*, *ZHX2*, *ZNF300*, and *ZNF710*) [24]. The *SH3PXD2B* gene found to have methylation at cg00718518 and gene expression signatures associated with trans-nonachlor in our study is a genome-wide association study (GWAS) locus for waist-hip ratio [27, 28], body weight [29], and balding measurement [27, 30]. Methylation at cg00718518 (*SH3PXD2B*) in liver tissue biopsies has been previously associated with obesity [31].

Mediation analysis revealed a potential explanation of the association between PBDE 47 and smaller birth weight through placental DNA methylation at *MAP4K5*. These findings support earlier studies that reported associations between POPs and birth weight [32] and a study in mice that highlighted activation of mitogen-activated protein kinases (MAPK) in the placenta of mice treated with PBDE 47 [33]. We also observed positive association between DNA methylation at another mitogen-activated protein kinase gene (*MAPK8IP1*) and PFUnDA. Both *MAP4K5* and *MAPK8IP1* placental DNA methylation levels were negatively correlated with birth weight in our study. Furthermore, PBDE 47 was associated with increased DNA methylation at cg04789362 (*KIAA0284*), and higher methylation at cg04789362 was associated with smaller weight and head circumference at birth. PBDE 47 was also associated with increased DNA methylation at cg01327147 (*KIAA1161*), and higher DNA methylation at cg01327147 was associated with decreased head circumference. Differential methylation at cg01327147 has been previously associated with neurodevelopmental syndromes [34]. The *KIAA1161* gene is known to play a role in brain calcification [35], abnormal cerebellum morphology, and functional neurological abnormalities related to dysfunction of the pyramidal tract [36]. Moreover, our mediation analysis suggested relations between PBDE 47 and head circumference via placental DNA methylation (cg01327147 (*KIAA1161)* and cg04789362 (*KIAA0284*)). Together, our study corroborates previous findings on the impact of maternal plasma POP concentrations and placental methylation, and adds to recent EWAS evidence for 15 novel placental methylation sites that could potentially impact placental function and fetal development [37].

We found strong corroborative evidence of exposure to PFUnDA on methylation and gene expression of the *TUSC3* gene, which is highly expressed in the placenta. In addition, placental methylation at cg18145877 (*TUSC3*) and expression of *TUSC3* gene was correlated with birth length. A published placental meQTL study [21] showed that sequence variants regulate methylation of cg18145877 of *TUSC3* in the placenta. *TUSC3* gene is a protein coding gene associated with several biological functions including cellular magnesium uptake, protein glycosylation, and embryonic development. *TUSC3* is a GWAS locus for mental health disorders and general cognitive ability including educational attainment and mathematical ability [38], obsessive-compulsive disorder [39], and schizophrenia [40]. In previous EWAS analyses, methylation at cg18145877 (*TUSC3*) in cord blood has been associated with prenatal arsenic exposure [41] and PCB 156 exposure [25]. PFUnDA was observed to be associated with decreased methylation at cg24722365 (*KIF1B*), and lower methylation at cg24722365 was associated with smaller birth length. *KIF1B* has been associated with body height in a published GWAS. In the same cohort, maternal plasma PFUnDA and PFHxS concentrations have been significantly associated with decreased neonatal thigh length at birth [8]. Studies have shown associations between prenatal exposure to PFAS and bone development [42, 43]. Our findings may point to potential pathway through placenta DNA methylation; however, the mediation analysis results were not significant.

Our study had several potential limitations that need to be considered in weighing our results. We are not aware of data from similarly-designed cohort studies, limiting our ability to replicate our findings in other independent populations. However, we were able to enrich the interpretation of our results using previous EWAS analyses involving biospecimens collected from adults or cord blood. There may be spatial and cell population-based differences in gene expression in the placenta, which was beyond the scope of our study. Our EWAS was able to identify modest methylation changes associated with maternal plasma POPs concentration, but we acknowledge that studies with larger sample sizes are needed to detect CpG sites with smaller methylation changes. The study was undertaken at 12 clinical sites that could potentially be related to differential methylation. However, in a sensitivity analysis evaluating models using the Akaike information criterion (AIC), 206 out of 214 differentially methylated CpG sites (96.3%) were better explained in models without than models with clinical sites, and all association *β* values were comparable (Supplementary Table S15). Finally, as chemicals mixture may be more complex than the sum of chemicals, we encourage future studies to further investigate the potential implication of interaction and non-linear relationships of POPs on placental epigenetic changes. Our study had several strengths. We adjusted our analysis for genotype-based principal components (PCs) in addition to methylation-based PCs, effectively minimizing spurious associations due to population stratification [44]. To date, there is no reference for cell type composition for placenta; therefore, we implemented a validated reference-free adjustment for cell type proportion variation implemented in SVA [45] and further corrected our analysis for genomic inflation using BACON, a method demonstrated to maximize study power while controlling for false discovery rate [46]. We also integrated our EWAS findings with placental gene expression and neonatal anthropometry measures to highlight potential mechanisms of prenatal POP toxicity through placental epigenetic changes.

## Conclusions

Findings from the present study suggest that maternal plasma concentrations of specific POPs may influence placental DNA methylation resulting in differences in neonatal birth size. Furthermore, we observed strong evidence consistently supporting a role for PFUnDA concentration on *TUSC3* including placental DNA methylation, gene expression, and placental meQTL analyses. Taken together, these findings shed light on potential placental epigenetic mechanisms that may explain associations between prenatal exposure to POPs and birth outcomes.

## Methods

### Study population

This analysis included 260 pregnant women from the NICHD Fetal Growth Studies–Singleton cohort—who provided placenta samples at delivery and had POPs concentration measures. Briefly, the NICHD Fetal Growth Studies–Singleton cohort included 2802 pregnant women enrolled between 8 weeks and 6 days and 13 weeks and 6 days between July 2009 and January 2013 from 12 clinic sites within the USA [47]. Eligible women could not have a past history of adverse pregnancy outcomes or self-reported behavioral risk factors such as use of cigarettes, illicit drugs, or alcohol in the months prior to pregnancy. The study was approved by institutional review boards at NICHD, all participating clinical and laboratory sites, and the data coordinating center.

### Environmental exposure data

For this ancillary study, all maternal blood samples used were collected at enrollment (10 weeks 0 days to 13 weeks 6 days) in phlebotomy equipment determined to be free of the POP contaminants under study. A total of 76 persistent organic pollutants (POPs) were measured, as described in a previous published paper [8]. We excluded chemicals where more than 20% of the concentrations were below laboratory LOQ. To capture the effect of the total exposure of each chemical class (i.e., OCPs, PCBs, PFASs), we summed individual chemicals within a class assuming additivity.

Briefly, PFASs were quantified using 200 μl of plasma, and PBDEs, PCBs, and OCPs were quantified using 1 ml of plasma. All samples were shipped in dry ice to the Wadsworth Center, New York State Department of Health [8]. Total plasma lipids were calculated using the short formula: total lipids (in ng/mL) = 2.27 * total cholesterol + triglycerides + 62.3 [48, 49], where total cholesterol and triglycerides (in nangrams per millimeters) were measured from non-fasting plasma stored plasma in − 80 °C freezers using the Roche COBAS 6000 chemistry analyzer (Roche Diagnostics, Indianapolis, IN) [50].

Machine-measured POP concentrations were modeled without substituting concentrations below the LOQ with a constant to minimize bias introduced when assessing health outcomes [51]. For analysis, POP concentration were *log* (*1 + chemical*) transformed for all POPs except for *p,p′*-DDE, PCB #138/158, sum of OCPs, and sum of PCBs where a *log* (*10 + chemical*) transformation was used. All concentrations were then rescaled by their standard deviations to provide results in interpretable units, i.e., change per 1 SD for each and summed POPs.

### Placental DNA methylation measurement and quality control

Placental samples (*n* = 312) were obtained within 1 h of delivery. Briefly, placental parenchymal biopsies measuring 0.5 cm × 0.5 cm × 0.5 cm were taken from the fetal side, placed in RNALater, and frozen for molecular analysis, as previously described [21]. Extracted DNA was assayed using Illumina’s Infinium Human Methylation450 Beadchip (Illumina Inc., San Diego, CA). Standard Illumina protocols were followed for background correction, normalization to internal control probes, and quantile normalization. Quality control procedures were followed as previously described [52]. Of the 301 pregnant women with placental DNA methylation data that passed quality filters, 260 women had POP concentrations available for analysis and represent the study cohort (260/312, 83.3%). For analyses, beta values were converted to the *M* value scale by using the formula: *M* value = log2(Beta/(1-Beta)). Any resulting infinity or missing *M* values (6.0%) were imputed by the *k-nearest* neighbors method, setting *k* = 10 for inclusion in the analysis [53].

### Placenta RNA quantification for gene expression

The placental biopsies used for DNA extraction were also used for extracting RNA from 80 placentas using TRIZOL reagent (Invitrogen, MA), and sequenced using the Illumina HiSeq2000 system. The expression of the transcripts were quantified using Salmon [54] which account for experimental attributes and biases such as fragment GC-content bias that is commonly observed in RNA-seq data. Participants with RNA-seq, DNA methylation, and POPs level at enrollment (*n* = 62) were used to test correlation between DNA methylation and gene expression levels.

### Statistical analysis

#### Epigenome-wide analyses

Epigenome-wide analyses were performed for each POP and each summed chemical class as the predictor and placenta DNA methylation at each CpG site as the outcome using the R/Bioconductor package “limma” [55]. Placental genome-wide SNP genotype data were used to estimate 10 genotype-based PCs representing the population structure. The R package “prcomp” was used to calculate PCs on the samples’ percent methylation profiles [56]. The EWAS analysis included robust linear regression models that were adjusted for self-reported maternal race/ethnicity (non-Hispanic White, non-Hispanic Black, Hispanic, Asian), age (in years), offspring sex (male/female), pre-pregnancy BMI (kg/m^2^), total plasma lipid concentration (ng/mL, except PFASs), log-transformed plasma cotinine level (ng/mL), methylation sample plate (*n* = 5), the first three methylation PCs and the first 10 genotype PCs to account for population structure [44], and putative cell-mixture estimated using *surrogate variable analysis (*SVA) components (*n* = 20) to account for latent source of noise such as batch effects and cell composition [57–59]. To account for the inflation of statistical test in EWAS, we implemented the Bayesian method to obtain BACON-corrected inflation estimates (Bλ) and BACON-corrected *p* values using the R/Bioconductor package BACON [46]. Corrected *p* values from BACON were then controlled for FDR [60] giving BACON-corrected FDR *p* values. Quantile-quantile plots of *p* values and the corresponding inflation estimates before (λ) and after (Bλ) BACON-correction are reported in Supplementary Figure S4. The inflation statistic was close to 1 after BACON correction (Supplementary Figure S4). Genome-wide Manhattan plots were used to report results from EWAS. The residual-vs-fitted plots for the first top hits of each EWAS are presented in Supplementary Figure S5.

To identify genomic regions that are differentially methylated with maternal plasma concentrations of POPs, we implemented DMR analysis using the R package dmrff [61]. Significant DMR was defined based on three criteria: (1) the DMR length can be at most 500 base pairs; (2) the region has EWAS FDR *p* values < 0.05, and (3) EWAS effect estimates for the CpGs have the same direction.

#### Gene expression analyses

We estimated the correlation between mRNA levels of the genes mapping to POP-associated CpG sites in our study (BACON-corrected FDR *p* values < 0.05) and placenta DNA methylation and corresponding maternal POPs blood concentration using Spearman correlations.

#### Canonical pathway analysis

Genes annotating to the epigenome-wide significant CpG sites (BACON-corrected FDR *p* values < 0.05) were further explored. We identified canonical pathway, networks, and diseases and biological function involved using the “Core Analysis” function in Ingenuity Pathway Analysis (IPA, QIAGEN Redwood City, CA, USA, www.qiagen.com/ingenuity). Diseases and function annotation with 3 molecules or more were considered. Statistical significance of overrepresented canonical pathways was determined using Fisher’s exact test after adjustment for multiple testing using the Benjamini-Hochberg method [60].

#### Functional annotation and analysis

To identify SNPs that may influence DNA methylation at the CpG sites in *cis* (i.e., within 1 megabase on either side of a gene’s transcript start site (TSS) [62]), we examined the significant CpG sites (BACON-corrected FDR *p* values < 0.05) in a list of SNPs that are known to be meQTL in the placenta from two previously published papers [21, 22].

Then the significant CpG sites (BACON-corrected FDR *p* values < 0.05) were queried in the mQTL database (http://www.mqtldb.org/) that documents methylation quantitative loci (meQTL) in human blood at serial time points across the life-course: birth, childhood, adolescence, middle age, and pregnancy [23]. Then, the *cis*-meQTL SNPs identified through the query were annotated using tools and databases including HaploReg v4.1 [63] and Genotype-Tissue Expression (GTEx) [64].

#### Correlations between top CpG sites and neonatal anthropometry measures

We examined whether maternal POP concentration and the methylation levels and gene expression level of the annotated gene for the significant differentially methylated CpG sites were correlated with neonatal anthropometry at birth (i.e., birth weight, length, and head circumference) using Spearman correlation coefficients.

#### Mediation analysis

Since maternal plasma POP concentrations were previously reported to be associated with neonatal anthropometry [8], we tested the possible mediation of the methylation at the CpG sites in the relation between the specific POP and neonatal anthropometry at birth using the R package “mediation” [65]. Mediation analyses were tested using the following criteria: (1) POP concentrations were associated with placental DNA methylation at the CpG site (BACON-corrected FDR *p* values < 0.05), (2) correlations between the CpG site and neonatal anthropometry measures had a *p* value < 0.10. The total effect represents the effect of the POPs on the neonatal anthropometric outcome without adjusting for methylation at the CpG sites; the direct effect represents the effect of the POP on the neonatal anthropometry measure at a fixed methylation level; the indirect effect represents the effect of the POP through the placental DNA methylation. Analyses were adjusted for self-reported maternal race/ethnicity (non-Hispanic White, non-Hispanic Black, Hispanic, Asian), age (in years), offspring sex (male/female), pre-pregnancy BMI (kg/m^2^), total plasma lipid concentration (ng/mL, expect PFASs), log-transformed plasma cotinine level (ng/mL), methylation sample plate (*n* = 5), the first three methylation PCs, and the first 10 genotype PCs.

## Supplementary information

**Additional file 1: Figure S1**. CpG sites relation to Island.

**Additional file 2: Figure S2**. Manhattan plots of associations between DNA methylation in placenta and maternal plasma concentrations of A – sum of OCPs, B- sum of PCBs, and C- sum of PFASs.

**Additional file 3: Figure S3**. Gene expression from the Human protein Atlas, consensus dataset.

**Additional file 4: Figure S4**. Quantile-quantile (QQ) plots with raw p-values and inflation estimates (λ) and with BACON-corrected p-values and BACON-corrected inflation estimates (Bλ).

**Additional file 5: Figure S5**. Residual-vs-fitted plots for the top hits of each EWAS. For each figure, one color represents one CpG site.

**Additional file 6: Supplementary Tables: Table S1**. Characteristics of the study subsample and of the full NICHD fetal Growth Studies – Singletons. **Table S2**: Description of the maternal plasma persistent organic pollutants concentrations. **TableS3**: Top-significant adjusted difference (BACON-corrected FDR p-values < 0.05) in placenta DNA methylation associated with maternal POP. **Table S4**: Differentially methylated regions (DMR) analysis. **TableS5**: Correlations between DNA methylation at the top differentially methylated CpG sites and gene expression in placenta. **Table S6**: Significant associations between maternal blood levels of POP and expression of genes near the top significant DNA methylation CpG sites. **Table S7**: Top 10 pathways of diseases and biological function from IPA. **TableS8**: IPA Canonical Pathway. **Table S9**: Network identified by Ingenuity Pathway Analysis. **Table S10**: Placental cis-eQTL analysis of top-significant (Bacon-adjusted FDR P-value<0.05) CpGs. **Table S11**: Cis-meQTL analysis of top-significant (Bacon-adjusted FDR P-value<0.05) CpGs. **Table S12**: Spearman correlation between top differentially methylated CpG sites and neonatal anthropometry. **Table S13**: Spearman correlation between neonatal anthropometry and gene-expression that were significantly correlated with the methylation on the corresponding CpG sites. **Table S14**: Comparison with previous EWAS on chemicals. **Table S15**: Sensibility analysis further adjusted for clinical sites for the CpG sites differentially methylated in the original model.

## Data Availability

The placental genome-wide DNA methylation, gene expression, and genotype data are available through dbGaP with accession number phs001717.v1.p1. The maternal genotype data analyzed in the current study are available from the corresponding author upon request. Summary statistics from the EWAS analysis are available as Supplementary Data.
