# Supplementary figures and images for: Concentrations of persistent organic pollutants in maternal plasma and epigenome-wide placental DNA methylation
Source: Clin Epigenetics. 2020 Jul 13;12:103. doi: 10.1186/s13148-020-00894-6 (PMC7371466; doi:10.1186/s13148-020-00894-6)

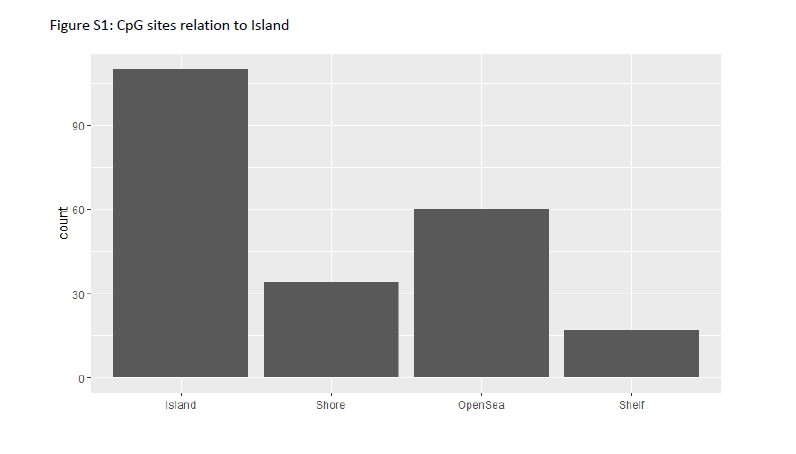

Supplement: Supplementary file 1 — Additional file 1: Figure S1. CpG sites relation to Island. [file 13148_2020_894_MOESM1_ESM.png]

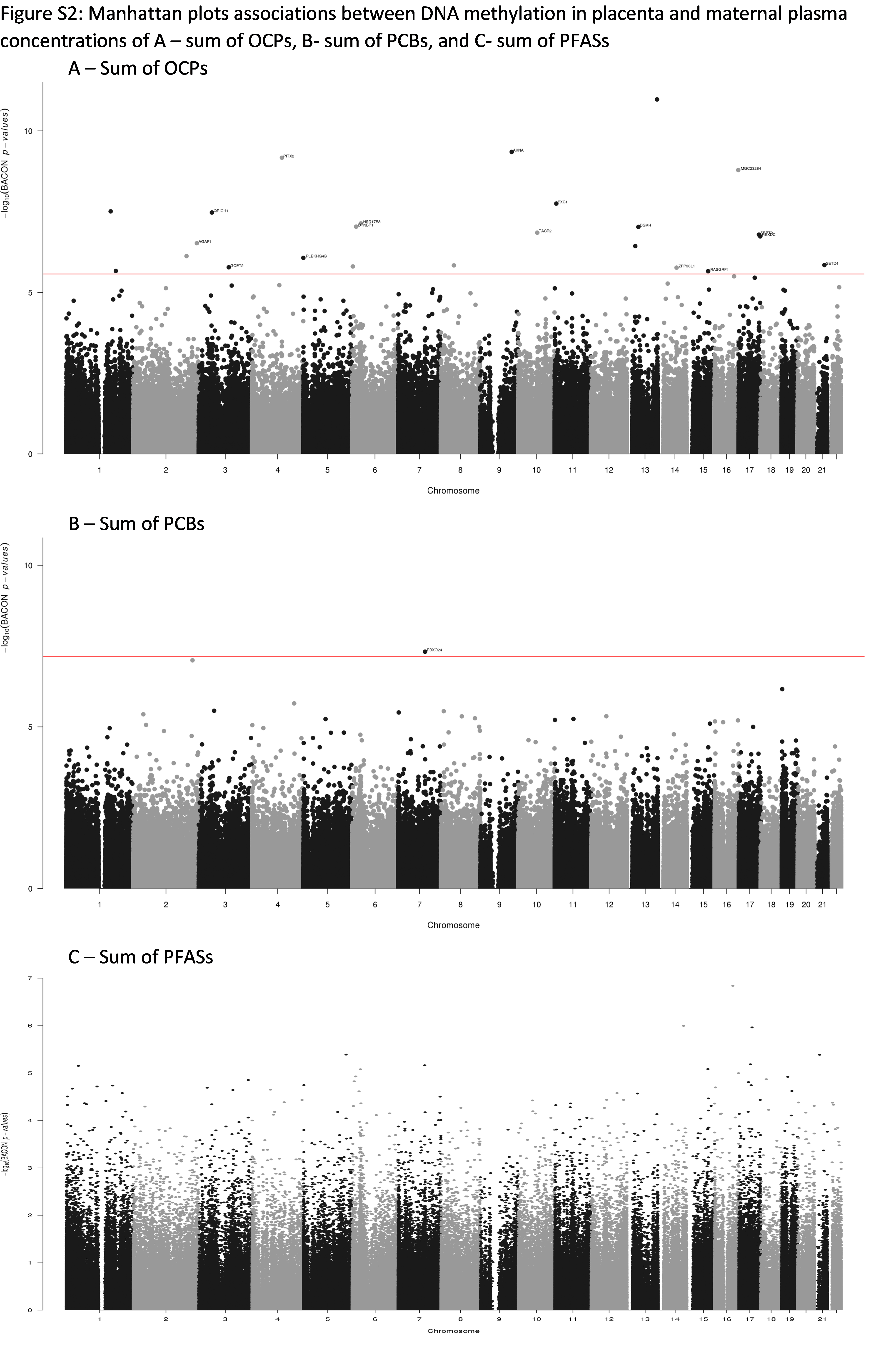

Supplement: Supplementary file 2 — Additional file 2: Figure S2. Manhattan plots of associations between DNA methylation in placenta and maternal plasma concentrations of A – sum of OCPs, B- sum of PCBs, and C- sum of PFASs. [file 13148_2020_894_MOESM2_ESM.png]

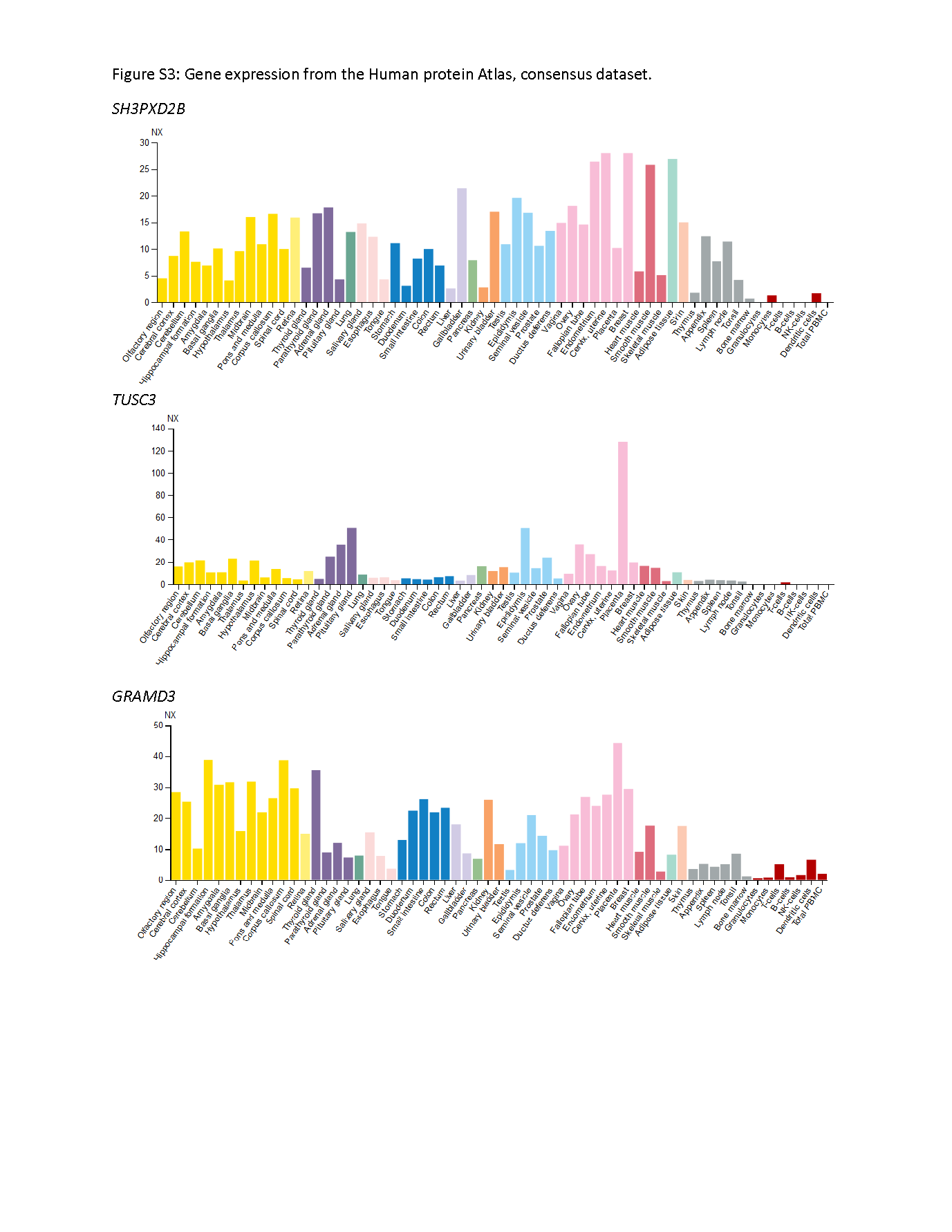

Supplement: Supplementary file 3 — Additional file 3: Figure S3. Gene expression from the Human protein Atlas, consensus dataset. [file 13148_2020_894_MOESM3_ESM.png]

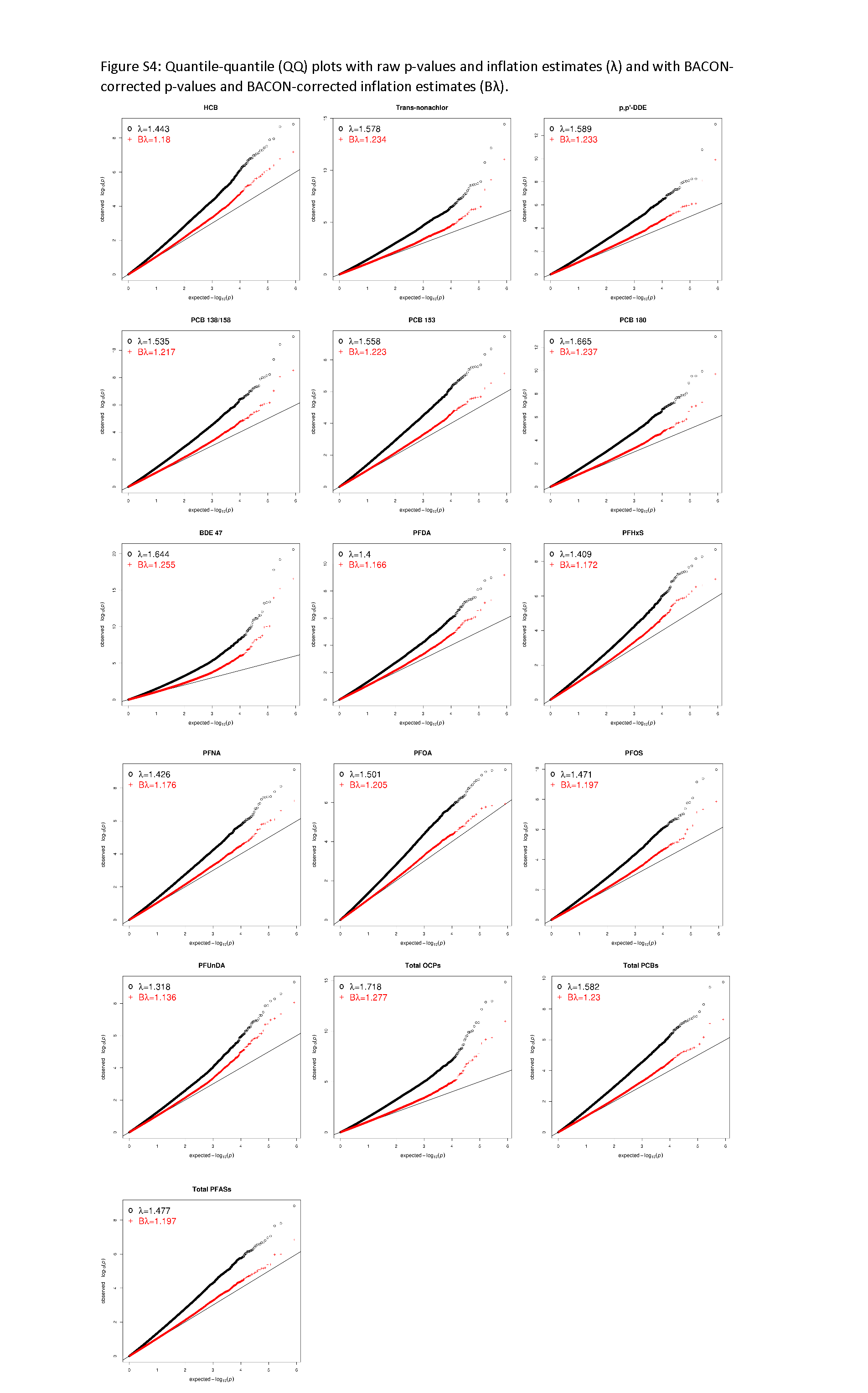

Supplement: Supplementary file 4 — Additional file 4: Figure S4. Quantile-quantile (QQ) plots with raw p-values and inflation estimates (λ) and with BACON-corrected p-values and BACON-corrected inflation estimates (Bλ). [file 13148_2020_894_MOESM4_ESM.png]

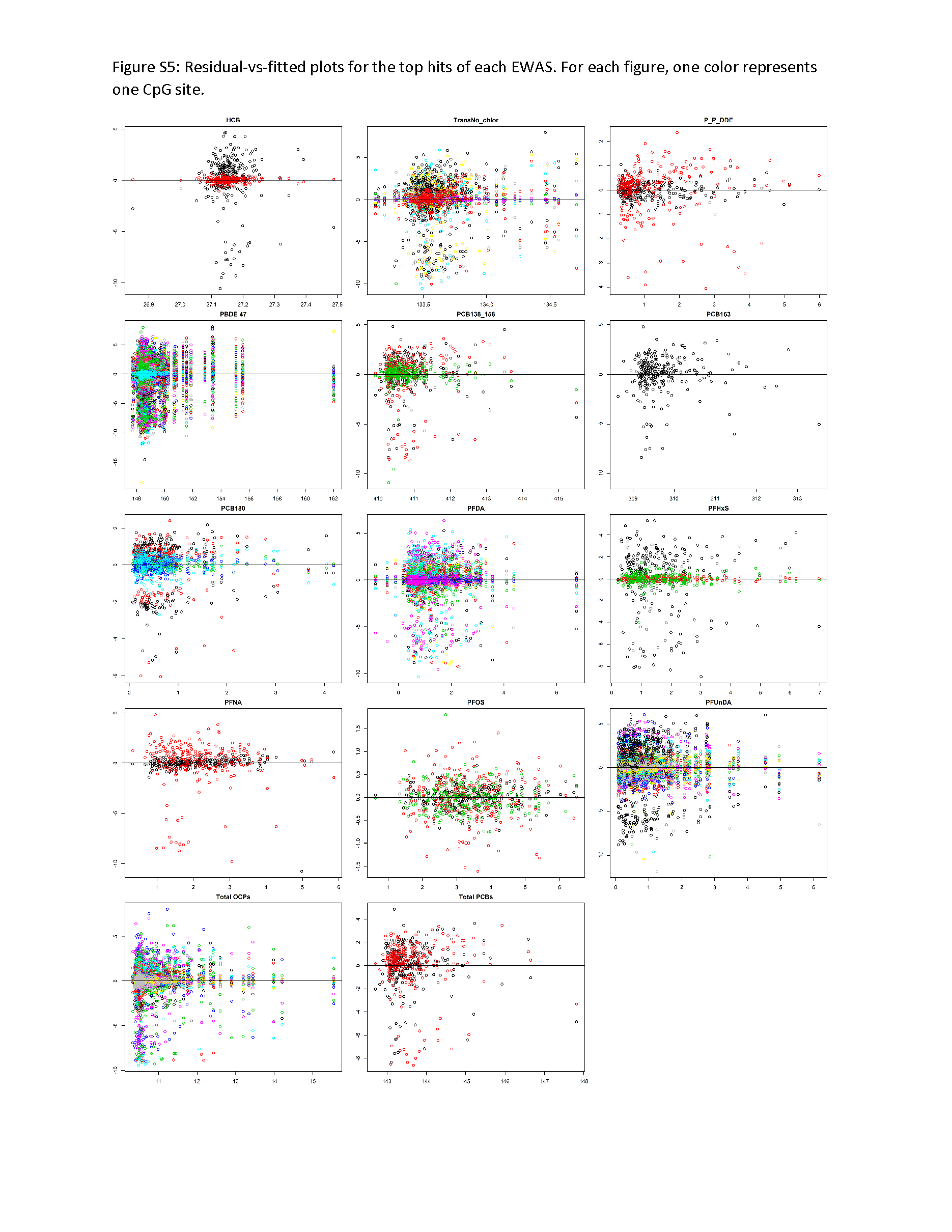

Supplement: Supplementary file 5 — Additional file 5: Figure S5. Residual-vs-fitted plots for the top hits of each EWAS. For each figure, one color represents one CpG site. [file 13148_2020_894_MOESM5_ESM.png]
